# A meta-analytic structural equation modeling analysis of the relationship between voice behavior, transformational leadership, and psychological capital

**DOI:** 10.3389/fpsyg.2026.1788980

**Published:** 2026-04-14

**Authors:** Yin-Che Chen, Yen-Ju Chen

**Affiliations:** Department of Educational Psychology and Counseling, National Tsing Hua University, Hsinchu City, Taiwan

**Keywords:** meta-analysis, psychological capital, structural equation modeling, transformational leadership, voice behavior

## Abstract

**Introduction:**

This study investigates the associations among voice behavior, transformational leadership, and psychological capital using a meta-analytic structural equation modeling approach. Drawing on conservation of resources theory, social identity theory, and broaden-and-build theory, psychological capital was proposed as a key mediator in the association between employee voice and transformational leadership.

**Methods:**

Data from 11 Taiwanese empirical studies published between 2008 and 2020 were synthesized, comprising more than 1,000 participants across diverse industries. Meta-analysis was conducted to examine the pairwise relationships among voice behavior, transformational leadership, and psychological capital, followed by structural equation modeling to test the proposed mediation model.

**Results:**

The meta-analysis revealed significant positive correlations between voice behavior and transformational leadership (*r* = .463), between voice behavior and psychological capital (*r* = .557), and between psychological capital and transformational leadership (*r* = .521). Structural equation modeling supported a mediation pattern in which psychological capital accounted for part of the positive association between voice behavior and transformational leadership, with the indirect effect being comparable to the direct effect.

**Discussion:**

These findings highlight the reciprocal nature of leader-follower dynamics and suggest that employee voice is systematically associated with employees’ psychological resources and with transformational leadership within the Taiwanese evidence base. This study contributes to the organizational behavior literature by clarifying the triangular relationship among voice behavior, psychological capital, and transformational leadership. The findings also have practical implications for fostering psychologically safe communication channels, investing in leadership development, and cultivating employee psychological capital to support organizational innovation and adaptability.

## 1 Introduction

Voice behavior helps organizations improve current practices, make higher quality decisions, correct errors, and strengthen processes, thereby fostering innovation (Burris, 2012; Fuller et al., 2007; Morrison, 2011; Wei et al., 2015). Given these benefits, employee voice has become a prominent topic in organizational research (Arham et al., 2022; Irshad and Naqvi, 2023; Liu et al., 2022; Su et al., 2017; Yan, 2018). Transformational leadership is likewise one of the most influential leadership perspectives in contemporary management research (Le and Lei, 2019). Prior studies have consistently reported a positive association between transformational leadership and employee voice (Chandra et al., 2022; Duan et al., 2017; Zhao and Luan, 2022). Transformational leaders attend to subordinates’ needs, stimulate innovative thinking, and build confidence and capability by demonstrating individualized concern, articulating vision, and infusing work with meaning (Yuan, 2014).

Recent empirical studies published in 2025 and 2026 have further underscored the relevance of integrating leadership, employee voice, and positive psychological resources. Specifically, inclusive leadership has been shown to promote employee voice through psychological capital and internal social capital (Yang et al., 2025a), leader voice endorsement transparency has been found to shape subsequent voice behavior (Ji et al., 2026), and shared leadership and servant leadership have each demonstrated significant associations with employee voice behavior (Bai and He, 2025; Hassan et al., 2025). In addition, recent studies have continued to link transformational leadership to employee voice, innovation related outcomes, and employees’ psychological resources across diverse organizational contexts (Jaafari et al., 2025; Kim and Yoon, 2025; Kuhlmann and Klingenberg, 2025; Yang et al., 2025b). Taken together, these findings suggest that leadership processes, employee voice, and positive psychological resources should be examined within a more integrated framework.

Despite extensive work treating leadership as an antecedent of voice, less attention has been devoted to the possibility that voice behavior may also be associated with leaders’ transformational practices. Voice can benefit employees by improving supervisors’ evaluations, enhancing work attitudes, and reducing turnover, and it can benefit organizations by improving innovation, decision making, and performance (Bashshur and Oc, 2015). These outcomes are broadly consistent with conditions associated with transformational leadership. Accordingly, employee voice expressed in employee leader interactions may be systematically related to leaders’ enacted leadership styles. This possibility may be especially salient in Confucian cultural contexts such as Taiwan, where employees may be more cautious about expressing dissent because such behavior can be interpreted as boundary crossing or as a challenge to authority (Chou et al., 2013; Wu and Ma, 2022). These cultural dynamics may shape both the frequency and expression of voice.

Engaging in voice is inherently demanding because employees must weigh possible responses and consequences before speaking up (Boudrias et al., 2020). Voice may yield formal rewards, such as pay increases, and informal rewards, such as higher status, but it may also generate interpersonal discomfort or tangible losses, including the risk of demotion (Bo et al., 2021). Conservation of resources theory (COR) suggests that effective coping and performance depend on the availability of valued resources (Hobfoll, 2001; Whitman et al., 2014). Resource gains may initiate gain spirals through reinvestment (Demerouti et al., 2004), whereas resource depletion may foster avoidance coping and emotional resource loss (Bentein et al., 2017). At the same time, organizations have increasingly emphasized employees’ positive psychological states as important antecedents of desirable work outcomes (Abbas and Raja, 2015). Within this broader perspective, psychological capital, comprising hope, self efficacy, optimism, and resilience, has received substantial scholarly attention and may contribute to organizational advantage beyond economic and human capital (Bergheim et al., 2015; Luthans et al., 2004).

Psychological capital reflects a positive psychological state of development, including hope, defined as persevering toward goals and redirecting pathways when necessary; self efficacy, defined as confidence in one’s ability to invest effort in challenging tasks; optimism, defined as positive attributions regarding present and future success; and resilience, defined as the capacity to rebound from adversity (Luthans et al., 2005). Positive psychology further suggests that such psychological resources support proactive behavior and reflect a positive and agentic orientation toward work and performance (Li et al., 2018; Luthans et al., 2005). In the context of employee voice, these psychological resources are theoretically important because they can buffer the interpersonal risk, uncertainty, and potential resource depletion associated with speaking up. Employees with higher psychological capital are more likely to persist in expressing constructive suggestions, frame concerns in a solution oriented manner, maintain confidence when encountering possible resistance, and recover from unfavorable reactions. Through these processes, psychological capital may help translate voice into more constructive employee leader exchanges and more positive relational conditions associated with transformational leadership. Because leadership can also be understood as a bidirectional influence process in which supervisors and employees mutually affect one another (Wang and Chen, 2003), employees’ psychological capital may contribute to a positive interpersonal climate and may be positively associated with leadership effectiveness and style (Avolio and Luthans, 2005). However, psychological capital has often been treated primarily as an outcome or predictor of individual performance, and its mediating role in linking employee behavior to leadership related outcomes remains underexamined. From this perspective, psychological capital is not only a positive employee resource but also a theoretically meaningful mechanism through which voice behavior may be associated with transformational leadership.

Methodologically, this study applies meta analytic structural equation modeling (MASEM), which integrates meta analysis and structural equation modeling (SEM) to address limitations commonly associated with single study designs, such as restricted samples and incomplete coverage of prior findings. By aggregating evidence across studies, MASEM enhances the generalizability of findings and enables simultaneous examination of the triangular relations among voice behavior, psychological capital, and transformational leadership. Although prior research has examined each pairwise association, relatively few studies have evaluated the full three variable structure within a single meta analytic framework.

Accordingly, the purposes of this study are threefold. First, this study synthesizes Taiwanese empirical evidence to examine whether voice behavior is positively associated with transformational leadership. Second, this study evaluates whether psychological capital constitutes a theoretically meaningful mechanism linking voice behavior and transformational leadership. Third, this study applies MASEM to test the triangular association structure among voice behavior, psychological capital, and transformational leadership within the Taiwanese context.

## 2 Theoretical framework

To provide a more coherent theoretical foundation, the present study is grounded primarily in COR, with broaden and build theory serving as a supplementary perspective for the linkage between psychological capital and transformational leadership. COR theory posits that individuals strive to obtain, retain, protect, and invest valued resources, that resource loss is more salient than resource gain, and that individuals with greater resources are better positioned to generate additional resources through gain spirals (Demerouti et al., 2004; Hobfoll, 1989, 2001; Whitman et al., 2014). This logic is particularly relevant to employee voice because speaking up requires employees to expend time, cognitive effort, emotional energy, and relational resources under uncertain interpersonal conditions (Bentein et al., 2017; Bo et al., 2021; Boudrias et al., 2020). Within the present framework, voice behavior is conceptualized as a form of resource investment, psychological capital as a core personal resource, and transformational leadership as a leadership condition associated with resource support, meaning, and development. On this basis, COR theory provides the primary logic for explaining the associations among the focal variables and for developing the mediation hypothesis.

### 2.1 Voice behavior and transformational leadership

Prior research consistently indicates a positive association between transformational leadership and employee voice (Adhyke et al., 2023; Ilyas et al., 2021; Wang et al., 2011). Leaders who display openness, support, and proactivity tend to increase employees’ willingness to speak up (Detert and Burris, 2007; Kim et al., 2019; Liu et al., 2010), and transformational leadership is therefore often treated as an antecedent of voice, frequently interpreted through social exchange or cost and benefit perspectives (Duan et al., 2017; Wang et al., 2019; Zhang and Inness, 2019). By contrast, fewer studies have examined whether voice behavior may also be associated with leaders’ transformational practices.

From a COR perspective, voice behavior is not a cost free act. It requires employees to invest personal resources under conditions of interpersonal uncertainty and possible relational risk. Traditional views cast leadership as a one way power relationship (McCrimmon, 1995; Zhang et al., 2014), whereas more recent work conceptualizes leadership as reciprocal and co constructed through ongoing interaction (Lin and Liao, 2015; Northouse, 2007; Uhl-Bien, 2006). When employees voice dissatisfaction or propose constructive change, they contribute information, initiative, and problem solving effort that may enrich leader follower exchanges and expand the resource base available for collective action (Svendsen and Joensson, 2016; Zhang et al., 2023). In COR terms, such voice may function as resource investment that improves communication quality, strengthens trust, and creates conditions conducive to leadership behaviors characterized by support, inspiration, and development.

This interpretation is also consistent with related research showing that goal directed actions communicate social signals such as trust and goodwill, thereby shaping relationship quality (Jain, 2022; Lindenberg, 2000). When subordinates speak up in ways that prioritize organizational benefit, leaders may interpret such behavior as a signal of commitment and cooperation, which can strengthen mutual trust and open communication (Hung and Tseng, 2012; Tsai, 2011). Relatedly, Duan et al. (2017) demonstrated that the Pygmalion effect mediates the relationship between transformational leadership and voice, suggesting that expectations and encouragement shape follower behavior. Extending this relational logic in the opposite direction, employee proactivity may likewise signal competence and constructive intent to leaders, thereby corresponding with stronger transformational leadership tendencies (Northouse, 2007). Because voice is often treated as an extra role behavior (Podsakoff et al., 1990; Van Dyne and LePine, 1998), it may foster resource rich conditions of trust, communication, and collaboration that are associated with transformational leadership.

The relevance of this relationship may be especially pronounced in Asian contexts. Voice research has been dominated by Western settings, which may have constrained how voice behavior and its relational meanings are conceptualized (Matsunaga, 2015). Recent work suggests that cultural features such as power distance and collectivism shape the relationship between voice and leadership in Asia Pacific settings (Wilkinson et al., 2020). For example, Kim and Ishikawa (2020) found that when teams had more mechanisms for expressing voice, leaders were more likely to display transformational leadership in Japan, South Korea, and China. In Confucian contexts, relational identification may encourage employees to view voice as fulfilling relationship based responsibilities, while leaders may interpret voice as loyalty and cooperation (Adhyke et al., 2023). Accordingly, the association between voice behavior and transformational leadership may reflect a reciprocal resource process that is underestimated in one directional leadership models.

*H1:* Voice behavior is positively associated with transformational leadership.

### 2.2 Voice behavior and psychological capital

Positive psychology shifted attention toward human strengths and positive psychological states (Seligman, 2002). Building on positive organizational behavior and the broader capital framework, Luthans and Youssef (2004) introduced psychological capital to organizational management as a personal resource that supports positive organizational behavior and constitutes a fourth form of capital alongside economic, human, and social capital (Cheng and Sung, 2021; Luthans et al., 2004). Individuals high in psychological capital tend to be more optimistic and confident (Luthans et al., 2007), and prior work has linked psychological capital to creativity, engagement, career achievement, coping, and lower stress and burnout (Fu and Charoensukmongkol, 2022; Yu et al., 2019). Psychological capital has also been associated with job satisfaction, wellbeing, healthy workplace relationships, and continuous learning (Jung and Yoon, 2015; Xu et al., 2022).

The association between voice behavior and psychological capital can be explained primarily through COR theory. COR proposes that individuals seek to acquire and protect valued resources and that resource gains can be reinvested to create gain spirals (Hobfoll, 1989, 2001). Although voice behavior can involve interpersonal and career related risks (Chou and Barron, 2016; Maynes and Podsakoff, 2014), it may also produce resource gains when employees contribute constructive suggestions, participate in problem solving, and experience recognition, efficacy, or a greater sense of impact (Carnevale et al., 2017; Cheng et al., 2016; Detert and Burris, 2007; Morrison, 2011; Mowbray et al., 2020; Wang et al., 2023). In this sense, voice may not only consume resources but also help build them. Employees who repeatedly engage in constructive voice may strengthen hope, optimism, confidence, and resilience through successful resource investment and feedback cycles.

Related research has also described voice as a form of organizational citizenship behavior oriented toward improving organizational functioning (Van Dyne and LePine, 1998). As a form of citizenship behavior, voice has been linked empirically to psychological capital (Avey et al., 2010b; Bergeron and Thompson, 2020; Chou and Barron, 2016). In addition, studies grounded in identity related perspectives suggest that when individuals define themselves as organizational members, they are more likely to speak up in ways that affirm their membership and contribution (Abrams and Hogg, 1988; Dutton et al., 1994; Tajfel and Turner, 1986). Although the present study does not use identity theory as a primary framework, these findings are compatible with the COR argument that resource investment through voice may reinforce positive self evaluation and resource accumulation. Overall, employees who engage in voice may, through repeated resource investment and constructive organizational participation, report higher psychological capital.

*H2:* Voice behavior is positively associated with psychological capital.

### 2.3 Psychological capital and transformational leadership

The relationship between psychological capital and transformational leadership is primarily compatible with COR theory and is further clarified by broaden and build theory. From a COR perspective, psychological capital represents a reservoir of personal resources that helps employees cope with demands, sustain motivation, and remain constructively engaged in leader follower interactions. Employees with greater hope, efficacy, optimism, and resilience may be better able to interpret challenging situations as manageable, respond positively to leader guidance, and maintain openness toward developmental influence. In resource terms, psychologically stronger employees are more likely to participate in and sustain interactions associated with transformational leadership.

Broaden and build theory offers a complementary explanation for this association by arguing that positive emotions broaden individuals’ thought action repertoires and build enduring psychological, cognitive, and social resources (Fredrickson, 2001). When employees possess stronger psychological capital, they may be more likely to internalize leaders’ vision, respond positively to individualized consideration, and engage more fully with transformational messages. Avey et al. (2010b) argued that individuals with stronger psychological resources are better able to recognize value in their experiences and may respond more strongly to transformational leadership. Conceptually, the components of psychological capital also align with major elements of transformational leadership, including individualized consideration, intellectual stimulation, motivation, and idealized influence.

The undoing effect further suggests that positive emotions can attenuate the physiological and psychological impact of negative emotions (Fredrickson et al., 2000). Applying this perspective to leadership, individuals with higher psychological capital may better withstand negative affect, remain receptive to transformational influence, and contribute to more positive leadership climates. Empirical studies have likewise reported a positive association between psychological capital and transformational leadership (Pires, 2017). Related evidence indicates that employees’ positive psychological states shape organizational atmosphere, effectiveness, and leadership style, and that employees high in psychological capital are more sensitive to transformational behaviors and more likely to report positive job attitudes (Lin, 2009; Tsai, 2000; Yuan, 2014). Organizations may also cultivate psychological capital to facilitate the implementation of transformational leadership (Mujib and Rosari, 2023). Taken together, COR theory and broaden and build theory both support the expectation that psychological capital will be positively associated with transformational leadership.

*H3:* Psychological capital is positively associated with transformational leadership.

### 2.4 The mediating role of psychological capital

Transformational leadership research has traditionally emphasized top down influence, whereby leaders shape followers through motivation and vision (Chen et al., 2018; Duan et al., 2017). More recent work, however, recognizes bottom up dynamics in which employees, as holders of meaningful resources, participate in shaping leadership processes through everyday interaction (Tripathi, 2021). Within this broader view, the mediating role of psychological capital can be explained through COR theory. When employees engage in voice, they invest personal resources, including time, emotional effort, and reputational capital. When such investment is met with acknowledgment, effectiveness, or constructive dialogue, it may generate resource gains that strengthen psychological capital, especially self efficacy, hope, optimism, and resilience (Hobfoll, 1989).

This logic is consistent with prior research showing that organizational citizenship behavior is associated with transformational leadership (Hapsari et al., 2021; Herlina and Saputra, 2021; Tjahjono and Rahayu, 2024), and that voice is often conceptualized as an extra role citizenship behavior (Bergeron and Thompson, 2020; Mohapatra et al., 2019). Voice promotes innovation and learning and may therefore contribute to the accumulation of personal resources, which in turn shape employee leader interactions. This process may be especially salient in Chinese cultural contexts, where relational orientation is central (Peng, 1999). Compared with Western role delineation, hierarchical relationships in Chinese organizations often extend into personal and social domains and involve emotional interaction and reciprocal obligation (Grant and Berry, 2011; Yan and Xiao, 2016). When subordinates receive support from supervisors, they may reciprocate through citizenship behaviors and work related support, reflecting relationship based exchange patterns (Zeng et al., 2024).

Accordingly, the proposed mediation model assumes that voice behavior is positively associated with psychological capital and that psychological capital is, in turn, positively associated with transformational leadership. In COR terms, voice represents resource investment, psychological capital represents accumulated personal resources, and transformational leadership reflects a relational context in which those resources are more likely to be reinforced and expressed. Broaden and build theory further supports this pathway by suggesting that positive psychological resources broaden employees’ receptivity to leaders’ care, vision, and developmental influence (Avey et al., 2010b; Fredrickson, 2001). On this basis, psychological capital is expected to account for part of the positive association between voice behavior and transformational leadership.

*H4:* Psychological capital mediates the positive association between voice behavior and transformational leadership.

### 2.5 Theoretical model

**Figure 1:**
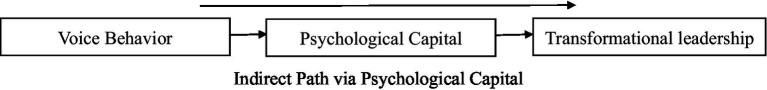
Theoretical model of the associations among voice behavior, psychological capital, and transformational leadership.

Figure 1 presents the theoretical model of the study. The model specifies a direct positive association between voice behavior and transformational leadership, together with an indirect pathway through psychological capital. Grounded primarily in COR theory, the model conceptualizes voice behavior as resource investment, psychological capital as a core personal resource, and transformational leadership as a relational and leadership condition associated with resource support and development. Broadly consistent with broaden and build theory, the model further assumes that positive psychological resources facilitate constructive engagement with transformational leadership.

## 3 Research method

### 3.1 Search strategy, data sources, and eligibility criteria

This study followed a systematic review logic consistent with the PRISMA checklist to identify, screen, and select studies for the meta analysis. The literature search focused on Taiwanese empirical research examining the associations among voice behavior, transformational leadership, and psychological capital. Because the purpose of the study was to synthesize evidence drawn specifically from the Taiwanese context, the search strategy was designed to maximize coverage of both peer reviewed publications and graduate theses that are frequently used in Taiwan based organizational research but are not always comprehensively indexed in international citation databases.

Three data sources were used: Google Scholar, Airiti Library, and the National Digital Library of Theses and Dissertations in Taiwan (NDLTD TW). Google Scholar was included because it provides broad interdisciplinary coverage and facilitates retrieval of journal articles, theses, conference related records, and other academic materials that may not be fully captured in a single commercial database. Airiti Library was used because it is a major repository of Taiwanese and Chinese language academic journals and therefore provides strong coverage of locally published management and organizational research. NDLTD TW was included because Taiwanese master’s theses and doctoral dissertations often report relevant quantitative findings, including correlation matrices and reliability coefficients, that are particularly valuable for meta analytic synthesis. Scopus and Web of Science were not used as primary search platforms because the present review targeted Taiwanese evidence specifically, and a substantial proportion of relevant local journals and dissertations are incompletely indexed or not indexed in those databases. Under these conditions, the selected databases were more appropriate for minimizing source undercoverage in the Taiwanese literature.

The literature search covered studies published between January 2008 and December 2020, corresponding to the period represented in the final body of evidence. Search terms were developed around the three focal constructs in both English and Chinese. The keyword combinations included “voice behavior” OR “employee voice,” “transformational leadership,” and “psychological capital,” together with their Chinese equivalents, including employee voice behavior, voice behavior, transformational leadership, transformational leadership, and psychological capital. These terms were searched both individually and in combination using Boolean operators adapted to the search syntax of each database. In addition, manual screening of reference lists and related records was conducted to identify potentially relevant studies that were not captured in the initial search.

Studies were eligible for inclusion if they met the following criteria: first, they examined at least one of the focal bivariate relationships among voice behavior, transformational leadership, and psychological capital; second, they used a Taiwanese sample; third, they employed a quantitative design that reported sufficient statistical information for effect size extraction, including Pearson correlation coefficients and sample size; and fourth, they reported acceptable measurement reliability. Studies were excluded if they reported only means and standard deviations without correlations, relied on recurring or overlapping samples, provided insufficient statistical information for effect size computation, or failed to meet minimum reliability requirements. Following this procedure, 75 records were initially identified, duplicates and ineligible reports were removed, and 11 studies were retained for the final meta analysis. The study identification and selection process is summarized in the PRISMA flow diagram.

### 3.2 Data extraction and coding procedure

A structured coding framework was used to extract the information required for both the pairwise meta analyses and the subsequent MASEM estimation. For each eligible study, the following information was recorded: author, year, publication type, sample size, participant characteristics, focal variables measured, measurement instruments, reliability coefficients, and the reported correlations among voice behavior, transformational leadership, and psychological capital. When a study reported only some of the focal correlations, the available coefficients were retained for the relevant pairwise synthesis.

Particular attention was given to data completeness and comparability. For dissertations and theses with limited accessibility, available materials, including abstracts, metadata, tables of contents, measurement descriptions, and statistical tables, were examined to verify variable definitions, sample size, reliability, and correlation coefficients. Only studies with sufficiently clear construct specification and extractable quantitative information were retained. In cases in which multiple reports appeared to rely on the same or highly similar samples, the report containing the more complete statistical information was retained in order to avoid double counting.

The extracted data were then organized by variable pair. Because not every study reported all three correlations required for a complete within study correlation matrix, the database for analysis consisted of partially overlapping subsets of studies. This feature of the evidence base informed the subsequent analytic strategy and required the pooled correlation matrix to be constructed from pairwise syntheses rather than from a complete common correlation matrix across all included studies.

### 3.3 Meta analytic procedure and MASEM specification

This study adopted a correlation based, two stage meta analytic structural equation modeling approach broadly consistent with Cheung (2008). In Stage 1, the three focal bivariate associations, namely the correlations between voice behavior and transformational leadership, between voice behavior and psychological capital, and between psychological capital and transformational leadership, were synthesized separately across the eligible Taiwanese studies. For each study, the reported Pearson correlation coefficient was transformed to Fisher’s *z* to stabilize the sampling distribution, and the corresponding sampling variance was defined as $v_{i}=1/(n_{i}-3)$ . Because the homogeneity tests indicated substantial between study heterogeneity, each pooled association was estimated under a random effects model. The study weight was calculated as $w_{i}=1/(v_{i}+\tau^{2})$ , where $\tau^{2}$ represents the between study variance component. The pooled Fisher’s *z* estimate was then computed as $\bar{z}=\sum (w_{i}z_{i})/\sum w_{i}$ and back transformed to *r* for substantive interpretation (Cheung, 2008).

Publication bias was assessed by combining visual inspection of funnel plots with fail safe N statistics for each pairwise synthesis. These procedures were used as diagnostic tools to evaluate whether the observed summary correlations were likely to be substantially distorted by nonreporting of nonsignificant findings.

In Stage 2, the pooled pairwise correlations were assembled into a synthesized correlation matrix and used as the input for structural equation modeling to evaluate the hypothesized mediation model. Because the number of contributing studies and the sample sizes differed across variable pairs, and because the primary studies did not consistently report a complete common correlation matrix, an effective sample size was required for SEM estimation. Following correlation based MASEM practice, the harmonic mean of the pair specific sample sizes was used as the working sample size in order to avoid allowing the largest contributing sample to dominate the precision of the structural estimates (Viswesvaran and Ones, 1995). Under this procedure, the structural path estimates, model fit indices, and bootstrap based indirect effects should be interpreted as theory consistent approximations derived from the pooled correlation matrix under incomplete matrix conditions, rather than as full information TSSEM estimates based on complete study level covariance structures.

### 3.4 Construction of the pooled correlation matrix

**Figure 2:**
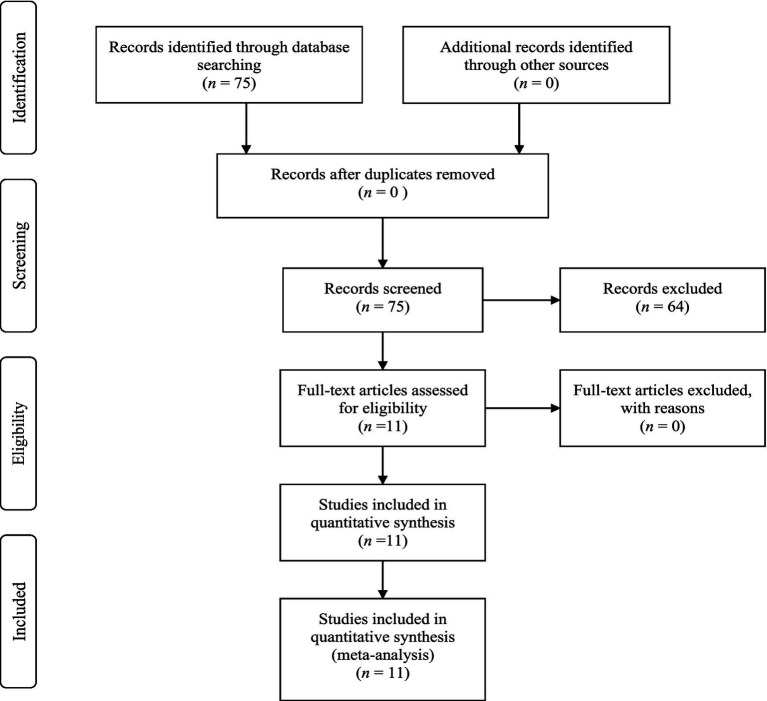
The flow diagram of PRISMA. PRISMA, Preferred Reporting Items for Systematic Reviews and Meta-Analyses.

The Stage 1 pooled correlations were assembled into a 3 × 3 meta analytic correlation matrix for the SEM stage, with 1.00 placed on the diagonal and the synthesized pairwise correlations entered into the corresponding off diagonal cells. This matrix represented the meta analytic association structure among voice behavior, psychological capital, and transformational leadership. Because the available evidence did not provide a complete within study covariance matrix for all focal variables across all studies, the present analysis relied on pairwise pooled correlations derived from partially overlapping samples. Accordingly, the resulting model should be understood as a constrained two stage MASEM application designed to evaluate the plausibility of the proposed triangular association structure under the reporting conditions of the Taiwanese literature (Figure 2).

## 4 Results

### 4.1 Relationship between voice behavior and transformational leadership

Three of the 11 studies reported the relationship between voice behavior and transformational leadership. A homogeneity test revealed that *Q* = 42.515 (*p* < 0.001), indicating statistically significant heterogeneity, and *I*^2^ = 95.296 indicated substantial between study variability. Under this degree of heterogeneity, the pooled coefficient should not be interpreted as a single population effect that can be generalized beyond the included evidence base. Rather, it is more appropriately understood as a common effect summary of the Taiwanese studies available for this relationship, retained for construction of the pooled correlation matrix used in the correlation based MASEM procedure (Cheung, 2008; Borenstein, 2009).

The pooled effect size was 0.463, and the 95% CI ranged between 0.399 and 0.522. The estimated effect size was converted to *Z* = 12.598 (*p* < 0.001), indicating a statistically significant positive association between voice behavior and transformational leadership.

**Figure 3:**
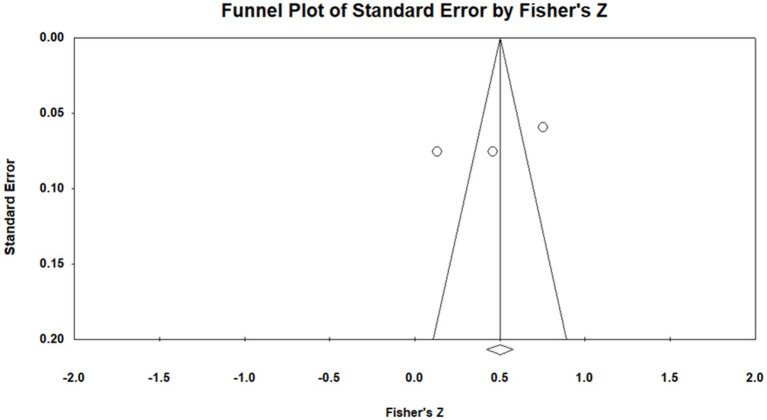
Funnel plot of analysis of relationship between voice behavior and transformational leadership.

Because the three selected studies included both dissertations and a journal article, publication bias was examined to reduce the risk of overestimating the pooled effect. According to the funnel plot (Figure 3), the three studies were positioned on both sides of the funnel, indicating no obvious publication bias. Moreover, Nf.s = 107, indicating that a total of 107 additional studies would be required for publication bias to nullify the observed result. The tolerance interval (5N + 10, with N representing the total number of samples in the meta analysis) was 25. According to Rosenthal (1991), when Nf.s exceeds the tolerance interval, unpublished or undiscovered statistically nonsignificant studies are unlikely to overturn the meta analytic result. Because Nf.s was larger than the tolerance interval, no publication bias was indicated for this relationship.

### 4.2 Relationship between voice behavior and psychological capital

Four of the 11 studies reported the relationship between voice behavior and psychological capital. A homogeneity test revealed that *Q* = 25.361 (*p* < 0.001), indicating statistically significant heterogeneity, and *I*^2^ = 88.171 indicated substantial between study variability. Accordingly, the pooled coefficient is best interpreted as a common effect summary of the available Taiwanese studies rather than as a population average effect generalized beyond the included evidence base (Cheung, 2008; Borenstein, 2009).

The pooled effect size was 0.557, and the 95% CI ranged between 0.518 and 0.593. The estimated effect size was converted to *Z* = 22.528 (*p* < 0.001), indicating a statistically significant positive association between voice behavior and psychological capital.

**Figure 4:**
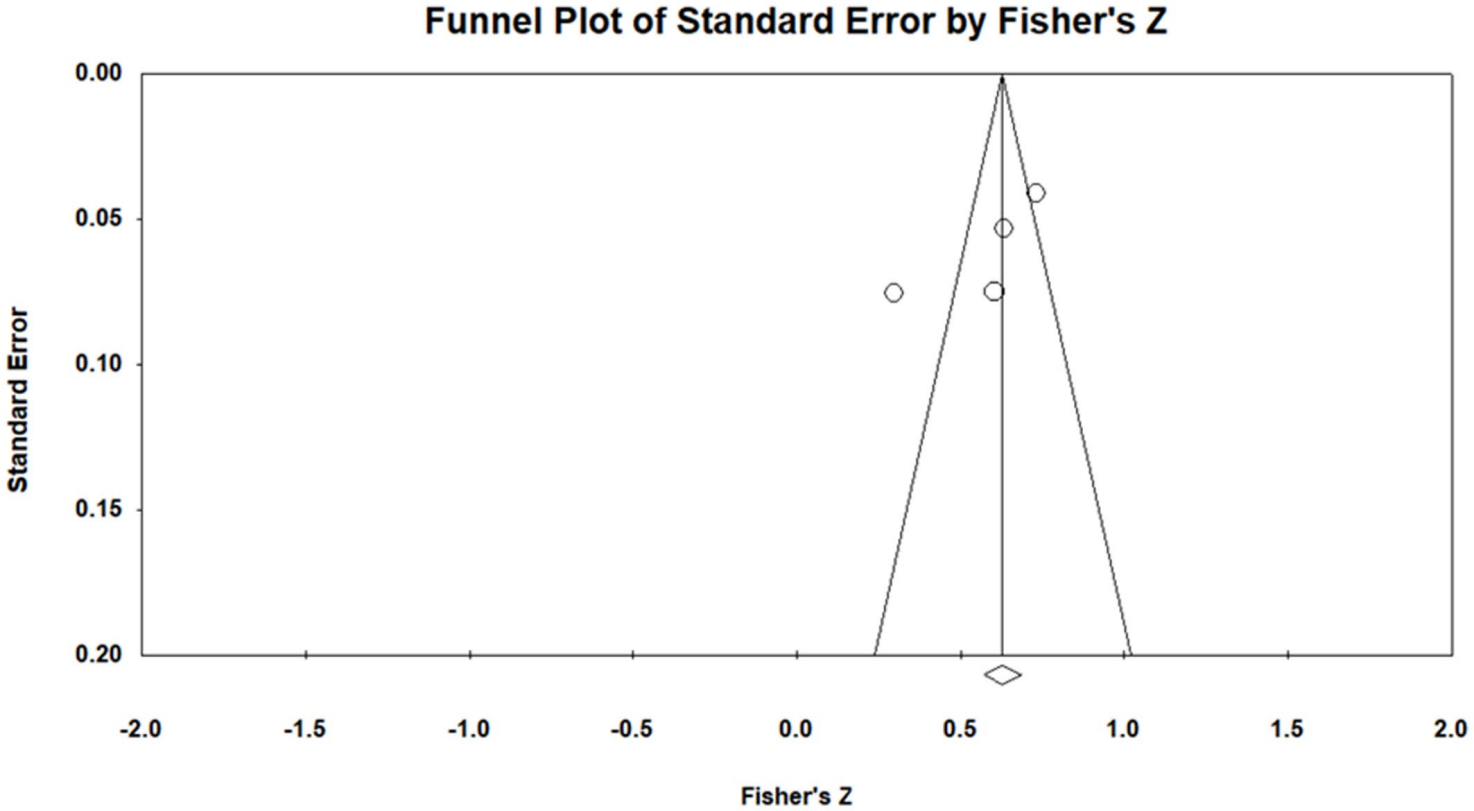
Funnel plot of analysis of relationship between voice behavior and psychological capital.

According to the funnel plot (Figure 4), the four selected studies were positioned on both sides of the funnel in a roughly symmetrical distribution pattern, indicating no obvious publication bias. Moreover, Nf.s = 445, indicating that a total of 445 additional studies would be required for publication bias to nullify the observed result. The tolerance interval (5N + 10) was 30, which was smaller than Nf.s. Accordingly, no publication bias was indicated for the relationship between voice behavior and psychological capital.

### 4.3 Relationship between psychological capital and transformational leadership

Six of the 11 studies reported the relationship between psychological capital and transformational leadership. A homogeneity test revealed that *Q* = 15.598 (*p* < 0.001), indicating statistically significant heterogeneity, and *I*^2^ = 67.944 indicated moderate to substantial between study variability. As in the preceding analyses, the pooled coefficient should therefore be interpreted as a summary of the included Taiwanese studies, not as an unrestricted population average (Cheung, 2008; Borenstein, 2009).

The pooled effect size was 0.521, and the 95% CI ranged between 0.476 and 0.563. The estimated effect size was converted to *Z* = 18.835 (*p* < 0.001), indicating a statistically significant positive association between psychological capital and transformational leadership.

**Figure 5:**
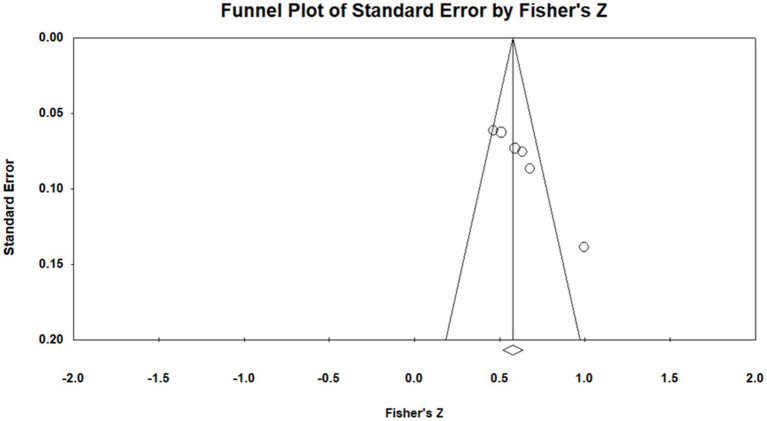
Funnel plot of analysis of relationship between psychological capital and transformational leadership.

According to the funnel plot (Figure 5), the six selected studies were positioned on both sides of the funnel in a roughly symmetrical distribution pattern, indicating no obvious publication bias. Furthermore, Nf.s = 572 indicated that a total of 572 additional studies would be required for publication bias to nullify the observed result. The tolerance interval (5N + 10) was 40, which was smaller than Nf.s. Accordingly, no publication bias was indicated for the relationship between psychological capital and transformational leadership.

### 4.4 Validating theoretical model of voice behavior, transformational leadership, and psychological capital

The correlation matrix among voice behavior, transformational leadership, and psychological capital was constructed for structural equation modeling, as shown in Table 1, and the sample sizes contributing to each pairwise correlation are reported in Table 2. The present analysis followed a constrained, correlation based, two stage MASEM approach. In Stage 1, the available pairwise meta analytic correlations were synthesized from the eligible Taiwanese studies and assembled into a pooled correlation matrix (Cheung, 2008; Hunter and Schmidt, 1990; Rosenthal, 1991). This procedure made it possible to integrate the available evidence and provide the correlation structure required for testing the hypothesized mediation model in the subsequent SEM stage.

**Table 1:** Correlation matrix between voice behavior, transformational leadership, and psychological capital.

Variable	Voice behavior	Transformational leadership	Psychological capital
Voice behavior	1.000		
Transformational leadership	0.463	1.000	
Psychological capital	0.557	0.521	1.000

**Table 2:** Sample size between voice behavior, transformational leadership, and psychological capital.

Variable	Voice behavior	Transformational leadership
Transformational leadership	642	
Psychological capital	1,298	1,082

In Stage 2, the synthesized correlation matrix was used as the input for structural equation modeling to evaluate the hypothesized mediation model. Because the pooled correlations were derived from unequal numbers of studies and unequal sample sizes, and because not all primary studies reported the complete set of correlations required for a full common matrix, the pooled matrix necessarily combined partially overlapping subsets of studies. Under these conditions, estimating the SEM stage with inconsistent pairwise sample sizes may produce nonpositive definite solutions or distort the precision of structural estimates. Accordingly, an effective sample size was required for model estimation. Following correlation based MASEM practice, the harmonic mean of the available pairwise sample sizes was adopted as the working sample size so that the largest contributing sample would not disproportionately determine the precision of the structural estimates (Viswesvaran and Ones, 1995). Specifically, the harmonic mean was 922 and was used as the overall sample size for SEM estimation.

Under this procedure, the standard errors of the structural paths were derived from the SEM estimation based on the pooled correlation matrix and the effective sample size, rather than from a full asymptotic covariance matrix of study level correlations. Therefore, the Stage 2 standard errors and model fit statistics should be interpreted as approximate indicators of model adequacy. This strategy is appropriate for evaluating the plausibility of the hypothesized triangular structure under incomplete matrix conditions, but it is more conservative to interpret the resulting SEM estimates as theory testing evidence rather than as fully exact multivariate meta analytic estimates. Accordingly, the structural model reported here should be understood as a synthesis of the available reported correlations under constrained evidence conditions, rather than as a full random effects TSSEM estimated from complete study level covariance matrices.

Table 3 presents the variable reliability analysis of the 11 selected studies. The average reliability, residual value, and square root of average reliability of each variable were calculated for SEM calculations.

**Table 3:** Summary of reliability of voice behavior, transformational leadership, and psychological capital.

Author	Voice behavior	Transformational leadership	Psychological capital
Huang (2018)	0.916		0.847
Lin (2018)	0.91		0.88
Shih (2018)	0.95		0.89
Chen (2016)	0.92	0.95	0.94
Chou et al. (2013)	0.89	0.95	
Tung (2020)	0.951	0.964	
Wu (2013)		0.99	0.97
Huang (2012)		0.922	0.862
Lin (2009)		0.946	0.915
Lai (2017)		0.96	0.98
Lu (2019)		0.97	0.89
Average reliability	0.923	0.957	0.908
Residual value	0.077	0.043	0.092
Square root of average reliability	0.961	0.978	0.953

### 4.5 Theoretical verification of relationship between voice behavior, transformational leadership, and psychological capital

#### 4.5.1 Basic goodness-of-fit criteria

**Figure 6:**
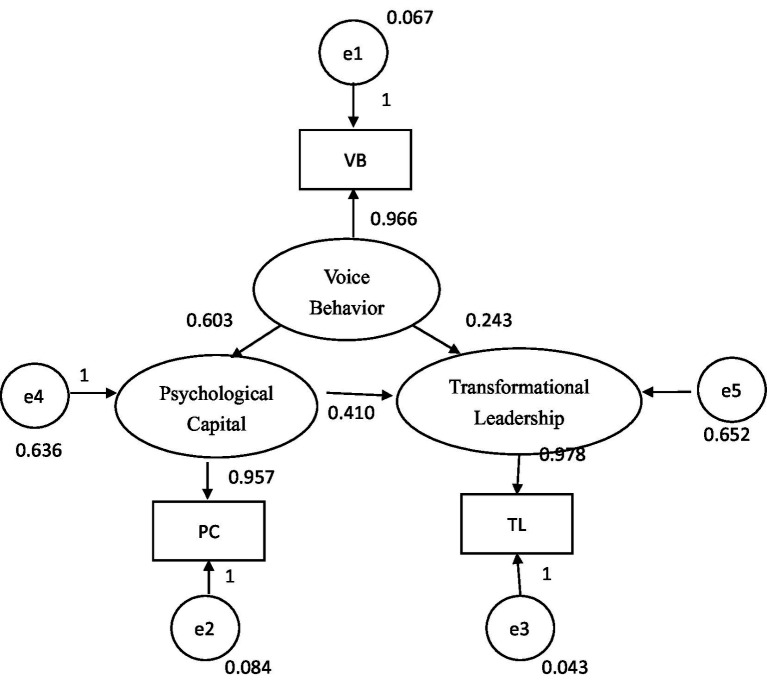
Path map and normalized parameter estimates of research model.

As shown in Figure 6, the parameter estimates of voice behavior to psychological capital, voice behavior to transformational leadership, and psychological capital to transformational leadership were all positive and <1, with the *p* values revealing statistical significance. Accordingly, these estimates fulfilled one of the basic goodness-of-fit criteria. The factor loadings of voice behavior, psychological capital, and transformational leadership were 0.966, 0.957, and 0.978, respectively, slightly higher than the threshold value of 0.95 (Bagozzi and Yi, 1988) while staying within the acceptable range. All the error term estimates were positive, with the standard deviations ranging from 0.033 to 0.050, fulfilling the recommendation by Bagozzi and Yi (1988) that standard deviations must not be excessively high. Accordingly, the parameters of the model did not exhibit poor estimates, and the quality of the model was satisfactory.

In sum, although the factor loadings of voice behavior, psychological capital, and transformational leadership were slightly higher than 0.95, the error variances of the three variables were positive and statistically significant. The absolute values between the estimated parameters were not close to 1, and the standard deviations were not excessively high. Accordingly, the theoretical model satisfied all the basic goodness-of-fit criteria and was accepted. Although the model fit indices (e.g., GFI = 1.00, SRMR = 0.00, CFI = 1.00) indicated excellent overall fit, these results should be interpreted with caution. The number of studies included in the meta-analytic correlation matrix was relatively small, which may artificially inflate fit indices (Cheung and Chan, 2005). The current findings offer preliminary support for the proposed model but should be further validated using larger and more heterogeneous datasets.

#### 4.5.2 Overall goodness-of-fit

The goodness of fit index of the research model was 1, higher than the threshold value of 0.9 and satisfying the absolute fit criterion. Both the root mean square residual and standardized root mean square residual were 0, satisfying conventional absolute fit criteria. The normed fit index, incremental fit index, and comparative fit index were all 1, indicating that the specified mediation structure was fully compatible with the pooled correlation matrix.

These fit statistics, however, should be interpreted cautiously. The model included only three observed variables and was estimated from a pooled matrix based on a limited number of studies, conditions under which near perfect fit indices can be produced more easily and may overstate model adequacy (Cheung and Chan, 2005; Cheung, 2008). The parsimonious normed fit index was 0 and did not satisfy the parsimonious fit criterion. Accordingly, the fit results support the plausibility of the hypothesized mediation structure within the synthesized Taiwanese evidence base, but they do not eliminate concerns related to heterogeneity, sparse study counts, or constrained model complexity.

#### 4.5.3 Internal structure fit

The individual reliability values of voice behavior, transformational leadership, and psychological capital were 0.933, 0.957, and 0.916, respectively, all of which were >0.5. The composite reliability values of the three variables were 0.870, 0.839, and 0.839, respectively, all of which were >0.6. The average variances extracted by the three variables were 0.870, 0.839, and 0.839, respectively, all of which were >0.5. Accordingly, the model adopted in this study exhibited satisfactory internal structure fit.

All the standardized residuals in the model were 0 (<1.96), also indicating that the model fulfilled satisfactory internal structure fit.

#### 4.5.4 Mediating effect of psychological capital on relationship between voice behavior and transformational leadership

The bootstrap method was performed 1,000 times to verify the mediating effect of psychological capital on the relationship between voice behavior and transformational leadership and to define the 95% CI accordingly (Table 4). An error-adjusted percentage bootstrap analysis and a conventional percentage bootstrap analysis were conducted, revealing that no zeroes existed in the 95% CI and that the *p* values indicated statistical significance. Accordingly, psychological capital substantially mediated the relationship between voice behavior and transformational leadership.

**Table 4:** Bootstrap verification of mediating effect of psychological capital.

Parameter	Method	Estimate	Lower limit	Upper limit	*p*
VB → PC → TL	Error-adjusted percentage bootstrap	0.247	0.194	0.293	0.003
	Conventional percentage bootstrap	0.247	0.198	0.295	0.002

VB, voice behavior; PC, psychological capital; TL, transformational leadership.

The total, direct, and indirect relations among voice behavior, transformational leadership, and psychological capital were then examined. The total effect of voice behavior on psychological capital (0.603) was identical to the direct effect, and the total effect of psychological capital on transformational leadership (0.410) was likewise identical to the direct effect. Accordingly, H2 and H3 were supported in terms of positive association, and no additional indirect relation was evident between voice behavior and psychological capital or between psychological capital and transformational leadership within the specified model. For the association between voice behavior and transformational leadership, the total effect (0.490) was the sum of the direct effect (0.243) and the indirect effect through psychological capital (0.247). Thus, the findings supported an indirect association pattern in which psychological capital statistically mediated the positive relationship between voice behavior and transformational leadership, supporting H4.

## 5 Discussion and suggestions

### 5.1 Discussion

#### 5.1.1 Relationship between voice behavior and transformational leadership

The results revealed that voice behavior was significantly and positively correlated with transformational leadership, with an effect size of 0.463 indicating a moderate association and a *Z* value of 12.598 (*p* < 0.001) confirming statistical significance. In substantive terms, higher levels of employee voice tended to co-occur with higher levels of transformational leadership within the synthesized Taiwanese studies. Relational signaling theory suggests that individuals adjust their behavior according to the situations they encounter and communicate goodwill through social signals, thereby fostering trusting and positive relationships with others (Jain, 2022; Moon et al., 2022). When employees engage in voice behavior for self-interest or for the benefit of the organization, such behavior may be associated with stronger organizational cooperation and more open communication. The Pygmalion effect has been discussed not only in relation to leaders’ influence on employees (Duan et al., 2017) but also in relation to how employees’ expectations and demands may be linked to leadership responses.

The results are consistent with existing research showing that voice behavior is significantly and positively associated with transformational leadership (Detert and Burris, 2007; Liu et al., 2010; Liu and Liao, 2013). According to Bashshur and Oc (2015), theoretical limitations and a lack of evidence have constrained understanding of how voice behavior relates to transformational leadership. The present findings contribute to this literature by clarifying the association between voice behavior and transformational leadership within the Taiwanese evidence base.

Recent empirical studies provide additional support for interpreting the present finding within a broader leadership and voice literature. Jaafari et al. (2025) reported that transformational leadership was positively related to employee voice and that this relationship operated through psychological empowerment, while Yang et al. (2025b) likewise showed that transformational leadership was associated with both employee innovation and voice in SMEs. Ji et al. (2026) further demonstrated that leader voice endorsement transparency shaped subsequent voice behavior, suggesting that leadership related signals concerning whether employee input is acknowledged and acted upon may be especially important for sustaining voice. At the same time, Bai and He (2025) showed that the relationship between leadership structures and voice may not be uniformly positive, as shared leadership can exert a “double edged sword” effect on employee voice behavior. Taken together, these studies reinforce the view that employee voice and leadership are embedded in an ongoing relational process. In this context, the present study contributes by showing that, within the Taiwanese evidence base, voice behavior is not only something that leaders may elicit, but is also positively associated with transformational leadership itself.

#### 5.1.2 Relationship between voice behavior and psychological capital

The results revealed that voice behavior was significantly and positively correlated with psychological capital, with an effect size of 0.557 indicating a moderate association and a *Z* value of 22.528 (*p* < 0.001) confirming statistical significance. In other words, higher levels of voice behavior tended to co-occur with higher levels of psychological capital. COR posits that individuals value and cultivate their own resources, continuously investing acquired resources into their work (Hobfoll, 1989, 2001). Thus, when employees engage in voice behavior, such behavior may be associated with addressing organizational issues and improving individual job performance (Carnevale et al., 2017; Mowbray et al., 2020), which in turn may coincide with higher psychological capital. Social identity theory further suggests that voice behavior can be understood as an extra-role form of organizational citizenship behavior (Bergeron and Thompson, 2020), through which employees identify themselves as part of the organization and act in ways intended to benefit it. In doing so, they may also strengthen their self-confidence and positive self-evaluations, which are consistent with higher psychological capital.

The present study examined the relationship between voice behavior and psychological capital through the lenses of COR and social identity theory. The findings supported H2 by indicating that voice behavior was positively associated with psychological capital. Chou and Barron (2016) argued that voice behavior is a type of organizational citizenship behavior, and Norman et al. (2010) indicated that it is positively associated with psychological capital. Avey et al. (2010b) likewise reported a positive relationship between psychological capital and voice behavior, and Jha (2021) contended that voice behavior is significantly and positively correlated with psychological capital.

Recent research also strengthens the interpretation of psychological capital as a relevant mechanism in the voice process. Yang et al. (2025a) found that inclusive leadership promoted employee voice through psychological capital and internal social capital, indicating that positive psychological resources are not merely peripheral conditions but meaningful pathways through which voice related behavior emerges. This evidence is consistent with the present result that voice behavior was positively associated with psychological capital. When considered together, these findings suggest that psychological capital may function both as an enabling condition for voice and as a resource that becomes reinforced through constructive employee participation. From a COR perspective, this pattern is theoretically important because it implies that voice may be associated with resource investment and resource gain at the same time, thereby supporting the argument that employees who speak up constructively may also report stronger hope, efficacy, optimism, and resilience.

#### 5.1.3 Relationship between psychological capital and transformational leadership

The results revealed that psychological capital was significantly and positively correlated with transformational leadership, with an effect size of 0.521 indicating a moderate association and a *Z* value of 18.835 (*p* < 0.001) confirming statistical significance. In substantive terms, higher levels of employees’ psychological capital tended to co-occur with higher levels of transformational leadership.

This result is consistent with the findings of Tsai (2000), who reported that psychological capital is significantly correlated with transformational leadership. Lin (2009) argued that employees with more abundant positive psychological capital are more sensitive to leaders’ transformational leadership behavior, which is associated with more favorable work attitudes. In a study of employees in China, Pires (2017) also found that psychological capital is significantly and positively correlated with transformational leadership. According to broaden-and-build theory (Fredrickson, 2001), positive emotions help individuals build psychological resources, creating a positive psychological cycle that increases receptivity to new challenges and innovative approaches. In the context of transformational leadership, leaders often engage employees through vision and inspiration. When individuals are in a positive emotional state, they may more readily perceive the potential value of transformational leadership, thereby constituting a complementary condition associated with such leadership (Avey et al., 2010b; Mujib and Rosari, 2023).

#### 5.1.4 Relationship between voice behavior, transformational leadership, and psychological capital

As indicated by the theoretical model, voice behavior, psychological capital, and transformational leadership exhibited a pattern of positive association consistent with the proposed framework. From the perspectives of COR and social identity theory, higher voice behavior was associated with higher psychological capital. Consistent with broaden-and-build theory, higher psychological capital was in turn associated with higher transformational leadership. Accordingly, the findings are more appropriately interpreted as supporting an indirect association pattern in which psychological capital statistically accounted for part of the positive association between voice behavior and transformational leadership, rather than as demonstrating a definitive causal sequence.

When employees identify more strongly with the organization through the process of social identification, they may place greater importance on organizational and personal interests simultaneously and experience pride in organizational membership (Swann and Bosson, 2010). According to COR, individuals may reinvest resources gained through work and thereby experience continuing resource accumulation and positive affect (Avey et al., 2010a). Broad-and-build theory further suggests that positive emotions broaden thought-action repertoires and build physiological, psychological, social, and cognitive resources. Accordingly, when employees possess positive psychological resources such as self-efficacy and optimism, they may be more receptive to leaders’ individual care and vision, and such receptivity may be associated with stronger transformational leadership within employee-leader interactions.

The present mediation pattern is also broadly consistent with recent studies examining psychological mechanisms in the leadership and voice literature. Yang et al. (2025a) showed that psychological capital mediated the relationship between inclusive leadership and employee voice, whereas Jaafari et al. (2025) identified psychological empowerment as a mediating mechanism linking transformational leadership and employee voice. Although these studies examined different directional paths and distinct mediators, they converge in suggesting that leadership and voice are not linked only at the behavioral level, but also through employees’ internal psychological resources. The present findings extend this line of work by indicating that psychological capital may statistically account for part of the positive association between voice behavior and transformational leadership. In this sense, the current study complements recent leadership research by locating psychological capital within a triangular association structure rather than treating it solely as a predictor or outcome of isolated dyadic relations.

In the present study, the factor loadings of the variables were slightly higher than 0.95 with respect to basic goodness of fit, and PNFI was lower than 0.05 with respect to overall goodness of fit. Nevertheless, the remaining basic and overall goodness-of-fit indices, including those related to internal structure fit, fulfilled their respective criteria, indicating that the theoretical model exhibited satisfactory fit. The mediating role of psychological capital was supported by bootstrap analysis, which showed that no zeroes were included in the 95% CI and that the total effect of voice behavior on transformational leadership (0.490) equaled the sum of the direct effect (0.243) and indirect effect (0.247). Accordingly, H4 was supported as a mediation hypothesis at the level of statistical association.

#### 5.1.5 Cross-cultural considerations

While the present findings are grounded in Taiwanese organizational contexts, they may not generalize directly to cultures with different values and workplace norms. For example, in high power distance cultures, such as many East Asian societies, employees may be less likely to express voice behavior openly (Chou et al., 2013; Wu and Ma, 2022), whereas in Western low power distance settings, voice behavior may be more normalized and expected (Morrison, 2011). Similarly, collectivist cultures may prioritize harmony over confrontation, thereby shaping both the form and the consequences of voice expression.

Previous studies in North America and Europe have shown that transformational leadership often encourages voice proactively (Detert and Burris, 2007), whereas the present study adds nuance by suggesting that, in Confucian cultural contexts, employee voice may also be positively associated with leadership behavior when it is expressed respectfully and constructively. This reciprocal pattern may be less visible in settings that place stronger emphasis on individual agency. Future research should therefore examine the model using cross-cultural MASEM to assess whether these associations remain stable across different national and cultural settings.

#### 5.1.6 Interpretation of effect sizes and practical significance

While the correlations among the key constructs were statistically significant, their effect sizes also warrant practical interpretation. For instance, the observed effect size of *r* = 0.463 between voice behavior and transformational leadership represents a moderate association (Cohen, 1988), suggesting that higher levels of employee voice behavior co-occur with meaningfully higher levels of transformational leadership. Likewise, the correlation between voice behavior and psychological capital (*r* = 0.557) indicates that voice behavior is closely associated with employees’ psychological resilience, hope, and confidence. These findings underscore the importance of creating organizational climates that encourage voice, as such climates may be associated with employee well-being and with adaptive leadership dynamics. In practice, interventions aimed at increasing voice behavior could include regular feedback loops, psychological safety training, and formal mechanisms for upward communication, all of which may help cultivate positive relational and psychological conditions in leadership processes.

#### 5.1.7 Theoretical implications

Beyond its practical implications, this study also offers several theoretical implications. First, the findings contribute to the literature on voice behavior and leadership by extending attention from the dominant top down perspective to a bottom up relational pattern. Prior research has primarily treated transformational leadership as an antecedent of employee voice (Detert and Burris, 2007; Duan et al., 2017; Liu et al., 2010). The present findings instead indicate that voice behavior is positively associated with transformational leadership and that this association can be partially accounted for by psychological capital. In this respect, the study broadens existing theorizing by showing that leadership related processes may also be linked to employee initiated resource investment and interaction patterns, rather than being explained exclusively by leader driven influence.

Second, the study contributes to conservation of resources (COR) theory by clarifying how voice behavior, psychological capital, and transformational leadership can be understood within a common resource based framework. COR theory posits that individuals seek to obtain, retain, protect, and invest valued resources, and that resource gains may accumulate through gain spirals over time (Hobfoll, 1989, 2001; Demerouti et al., 2004). The present findings are consistent with this logic in three ways. Voice behavior can be understood as a form of resource investment because speaking up requires time, cognitive effort, emotional regulation, and relational risk management under uncertain interpersonal conditions (Bentein et al., 2017; Bo et al., 2021; Boudrias et al., 2020). Psychological capital can be understood as a core personal resource that reflects employees’ capacity to sustain effort, remain optimistic, and recover from setbacks (Luthans et al., 2005). Transformational leadership, in turn, can be understood as a relational context associated with support, meaning, and developmental reinforcement. By linking these constructs, the present study extends COR theory from a general stress and coping perspective to a more explicit explanation of how employee initiated behavior may be associated with resource accumulation and leadership dynamics in organizational settings.

Third, the mediating role of psychological capital offers a more specific theoretical contribution to COR. Rather than treating psychological capital only as an antecedent or outcome, the present findings position it as a resource mechanism that may account for part of the positive association between voice behavior and transformational leadership. This perspective is theoretically important because it suggests that the consequences of voice are not limited to external outcomes, such as supervisor evaluation or organizational change, but may also operate through the development and activation of employees’ internal psychological resources. In COR terms, voice behavior may be associated with resource gain when employees experience efficacy, recognition, and constructive exchange, and these resource gains may correspond with stronger transformational leadership perceptions and interactions. This interpretation adds theoretical precision to COR by illustrating how personal resources may connect employee behavior with leadership related outcomes.

The recent literature also highlights why the present theoretical contribution remains timely. Bai and He (2025) suggested that leadership arrangements can have mixed implications for voice, indicating that voice related dynamics are more complex than a simple uniformly positive leadership effect. Ji et al. (2026) further showed that leader endorsement transparency influences subsequent voice behavior, underscoring the importance of relational interpretation and resource signaling in voice processes. In addition, Kim and Yoon (2025) and Yang et al. (2025b) found that transformational leadership remained closely linked to innovative and proactive employee outcomes in technology and SME contexts, while Yang et al. (2025a) demonstrated that psychological capital continues to serve as a meaningful explanatory mechanism in contemporary voice research. Viewed together, these newer studies support the present argument that a resource based perspective is useful for integrating leadership, employee voice, and positive psychological states within a common framework.

Fourth, the study contributes to COR by situating resource processes within a culturally specific context. The Taiwanese setting is theoretically meaningful because voice behavior in Confucian and relatively higher power distance contexts may involve greater interpersonal caution and stronger relational considerations (Chou et al., 2013; Wu and Ma, 2022). Under such conditions, the resource demands of speaking up may be especially salient, making COR theory particularly relevant for explaining why psychological capital matters. The findings therefore suggest that COR may be useful not only for explaining resource processes in general organizational behavior, but also for understanding how resource investment and resource protection operate in contexts where hierarchy, harmony, and relational sensitivity shape employee leader interactions.

Taken together, these findings contribute to theory by clarifying the triangular association among voice behavior, psychological capital, and transformational leadership, by strengthening the explanatory role of COR in this domain, and by showing that leadership related dynamics can be interpreted through a resource based framework that incorporates both employee action and psychological resource development.

### 5.2 Practical implications

The findings of this study offer several actionable recommendations for practitioners seeking to enhance transformational leadership and promote employee voice behavior through the development of psychological capital.

First, organizations should establish psychologically safe and structured communication channels—such as anonymous suggestion systems, dedicated voice forums, and regular dialogue sessions between employees and managers. These mechanisms help reduce perceived interpersonal risk and encourage upward communication, which in turn fosters constructive feedback loops (Detert and Treviño, 2010; Morrison, 2014). Recent evidence suggests that post-pandemic workplace shifts have increased the importance of formal voice channels in sustaining employee engagement and innovation.

Second, investment in leadership development is essential, particularly programs that cultivate emotional intelligence, active listening, and inclusive leadership practices. These competencies have been shown to improve team cohesion, trust, and transformational outcomes (Harms and Credé, 2010; Wang et al., 2021). In hierarchical or bureaucratic contexts, equipping mid-level managers with transformational skills can strengthen the vertical communication chain and enhance responsiveness to employee voice (Carnevale et al., 2017).

Third, organizations should adopt targeted human resource development initiatives to build employees’ psychological capital. Evidence from recent interventions demonstrates that resilience training, strengths-based coaching, and mindfulness programs can significantly boost self-efficacy, optimism, and resilience, thereby improving both well-being and job performance (Luthans et al., 2006; Avey et al., 2011). These programs are particularly valuable in high-stress service industries, such as healthcare and hospitality, where emotional labor is intensive.

Fourth, formal integration of voice behavior into performance evaluation systems can help institutionalize it as a valued organizational norm. Research shows that when constructive input is recognized and rewarded—through promotions, bonuses, or informal recognition—employees are more likely to engage in proactive voice behavior, which in turn stimulates leaders’ transformational behaviors (Liang et al., 2012).

Fifth, industry-specific tailoring of voice and leadership initiatives enhances their effectiveness. In innovation-driven sectors such as technology and R&D, embedding voice norms into agile team processes can accelerate product development and organizational learning (Kwon and Farndale, 2020). Conversely, in more hierarchical sectors, structured and protected voice mechanisms may be required to overcome cultural barriers to speaking up (Kim and Ishikawa, 2020).

Sixth, implementing transparent reward systems can reinforce employees’ motivation to speak up. Leaders should model openness by visibly acting upon employee suggestions and providing feedback on how voice inputs influence decision-making. Such leader responsiveness signals respect and reciprocity, strengthening both commitment and trust (Ng and Feldman, 2015; Zhu et al., 2022).

Finally, strategically deploying employees with high psychological capital into key roles—such as project coordination or change management—can maximize organizational adaptability. High-PC employees tend to be more resilient, proactive, and responsive to leadership, thereby accelerating the diffusion of transformational practices across teams (Avey et al., 2011).

Collectively, these strategies may help create conditions in which psychological safety, voice behavior, psychological capital, and transformational leadership mutually reinforce one another, thereby supporting sustained organizational growth and adaptability.

### 5.3 Research limitations

This meta analysis is subject to several limitations. First, the included studies varied in measurement approaches, including scale selection, item content, participant characteristics, and scoring criteria. Because the available evidence base was limited, the present analysis was unable to further classify instruments or test moderators associated with scale type, industry, occupation, or demographic composition. As a result, measurement related error and contextual variation may remain embedded in the pooled estimates.

Second, substantial between study heterogeneity was observed across all three pairwise syntheses. Specifically, all *Q* statistics were statistically significant, and the corresponding *I*^2^ values ranged from 67.944 to 95.296, suggesting considerable variability across studies. Under such conditions, the pooled coefficients are more appropriately interpreted as conditional summaries of the included Taiwanese studies than as stable population averages that can be generalized to other settings (Borenstein, 2009; Cheung, 2008). Although a larger evidence base would allow random effects or mixed effects MASEM with moderator analysis to model such heterogeneity more directly, the current evidence base did not support that level of analysis.

Third, an additional limitation concerns the level of information available for MASEM estimation. A full information TSSEM requires complete study level correlation matrices together with their associated sampling covariance structure, yet many of the included studies reported only selected bivariate correlations. Consequently, the present analysis relied on pairwise pooled correlations to construct the pooled correlation matrix and used an effective sample size for SEM estimation. More specifically, the structural model was estimated from partially overlapping subsets of studies, and the harmonic mean of sample sizes was adopted as a conservative working N (Viswesvaran and Ones, 1995).

Although this strategy permits theory driven synthesis when complete matrices are unavailable and makes it possible to examine the hypothesized triangular relations, it does not fully capture the dependency among correlations estimated from the same primary study, nor does it eliminate the inferential constraints associated with missing correlations, limited study counts, or constrained model specification. Future research should therefore collect complete covariance matrices and implement a full random effects TSSEM or one stage MASEM framework in order to obtain more precise multivariate standard errors and model tests (Cheung, 2008).

Finally, the study focused exclusively on Taiwanese evidence. This focus is theoretically meaningful given the cultural relevance of voice behavior and leader follower relations, but it also limits the external validity of the findings across other national and organizational contexts. Comparative and cross cultural MASEM would be valuable for determining whether the indirect pathway from voice behavior to transformational leadership through psychological capital remains stable across differences in power distance, collectivism, and industry conditions.

## 6 Conclusion

The meta-analysis revealed that all the *Q* values on the relationships between voice behavior, transformational leadership, and psychological capital attained statistical significance, indicating that the relationships between all the variables were heterogeneous. All the effect sizes of the variable relationships were ≥0.45, with 95% CI not containing zeroes and reaching statistical significance, showing that the variables were moderately correlated with each other.

The effect size between voice behavior and transformational leadership was 0.463, with the lower and upper limits being 0.399 and 0.522 in a 95% CI, respectively, and containing no zeroes. Accordingly, the effect size was statistically significant. The *Z* value of 12.598 was also statistically significant, supporting H1; that is, voice behavior was significantly and positively correlated with transformational leadership.

The effect size between voice behavior and psychological capital was 0.557, with the lower and upper limits being 0.518 and 0.593 in a 95% CI, respectively, and containing no zeroes. Accordingly, the effect size was statistically significant. The *Z* value of 22.528 was also statistically significant, supporting H2; that is, voice behavior was significantly and positively correlated with psychological capital.

The effect size between psychological capital and transformational leadership was 0.521, with the lower and upper limits being 0.476 and 0.563 in a 95% CI, respectively, and containing no zeroes. Accordingly, the effect size was statistically significant. The *Z* value of 18.835 also was statistically significant, supporting H3; that is, psychological capital was significantly and positively correlated with transformational leadership (see Table 5).

**Table 5:** Meta-analysis of correlations between variables.

Variable	Number of studies	Effect size	95% CI		*Z*	Test of homogeneity		5N + 10
			Lower limit	Upper limit		*Q*	*I* ^2^	
Voice behavior vs. transformational leadership	3	0.463	0.399	0.522	12.598***	42.515***	95.296	25
Voice behavior vs. psychological capital	4	0.557	0.518	0.593	22.528***	25.361***	88.171	30
Psychological capital vs. transformational leadership	6	0.521	0.476	0.563	18.835***	15.598**	67.944	40

***p < 0.001; **p < 0.01; *p < 0.05.

As reported in Table 5, the calculated fail-safe N values were substantially higher than the threshold value of 5k + 10 for all three correlations examined. For instance, the fail-safe N for the voice behavior–transformational leadership correlation was 107, well above the threshold of 25. This suggests that it would require over 100 unpublished null-result studies to reduce the observed effect size to a non-significant level, providing robust support for the absence of publication bias.

The bootstrap method was performed 1,000 times to verify the mediating role of psychological capital in the relationship between voice behavior and transformational leadership, and the 95% CI contained no zeroes. The total effect of voice behavior on transformational leadership (0.490) was the sum of the direct effect (0.243) and the indirect effect (0.247), supporting H4. These findings support a statistically significant indirect association between voice behavior and transformational leadership through psychological capital, rather than a definitive causal pathway.

## 7 Suggestions for future research

Because the included studies varied in the scales, item content, and measurement criteria used to assess the same constructs, the limited number of available samples prevented us from categorizing instruments and testing whether results differed by scale type. We also could not examine demographic and contextual characteristics within each study, such as gender, age, industry, organizational type, or job seniority. Future research should compile a sufficiently large evidence base to enable subgroup and moderator analyses that clarify whether effect estimates vary systematically across measurement approaches and sample profiles.

This study synthesized questionnaire based findings and evaluated the proposed relationships using a quantitative SEM framework. To enhance interpretive depth and strengthen construct validation, future work could incorporate qualitative approaches, such as interviews, observations, or textual analyses of existing qualitative materials. Triangulating qualitative and quantitative evidence would provide richer insight into how voice behavior and psychological capital operate in day to day employee leader interactions and how these processes translate into transformational leadership.

Finally, the present model was restricted to voice behavior, psychological capital, and transformational leadership, leaving other theoretically relevant variables unexamined. Future meta analytic work can extend this framework by incorporating additional individual and organizational level factors, conducting expanded goodness of fit tests, and improving explanatory power, particularly given the individual level nature of voice behavior and psychological capital and the organizational level framing of transformational leadership. In addition, cross cultural replications are needed to assess whether the mediating role of psychological capital and the reverse pathway from voice behavior to leadership vary across cultural dimensions such as power distance and collectivism, potentially using comparative meta analyses or multi group MASEM. Stronger causal inference would also benefit from experimental and longitudinal designs, including vignette or field experiments that manipulate psychological capital and panel studies that track the temporal sequencing and reciprocity between voice and leadership over time.

## Data Availability

Publicly available datasets were analyzed in this study. This data can be found at: the datasets analysed for the current study are available from the corresponding author upon reasonable request.
